# Supplementary material for: Capicua deficiency induces autoimmunity and promotes follicular helper T cell differentiation via derepression of ETV5
Source: Nat Commun. 2017 Jul 12;8:16037. doi: 10.1038/ncomms16037 (PMC5510180; doi:10.1038/ncomms16037)
Supplement: Supplementary Information [file ncomms16037-s1.pdf]

File name: Supplementary Information
Description: Supplementary Figures

File name: Supplementary Data 1
Description: Differentially expressed genes (DEGs) in naïve and anti-CD3/CD28 antibody-activated Cic deficient CD4+ T cells.

File name: Supplementary Data 2
Description: Primer sequences used in ChIP experiments

File name: Supplementary Data 3
Description: Primer sequences used in qRT-PCR experiments

File name: Peer Review File
Description:

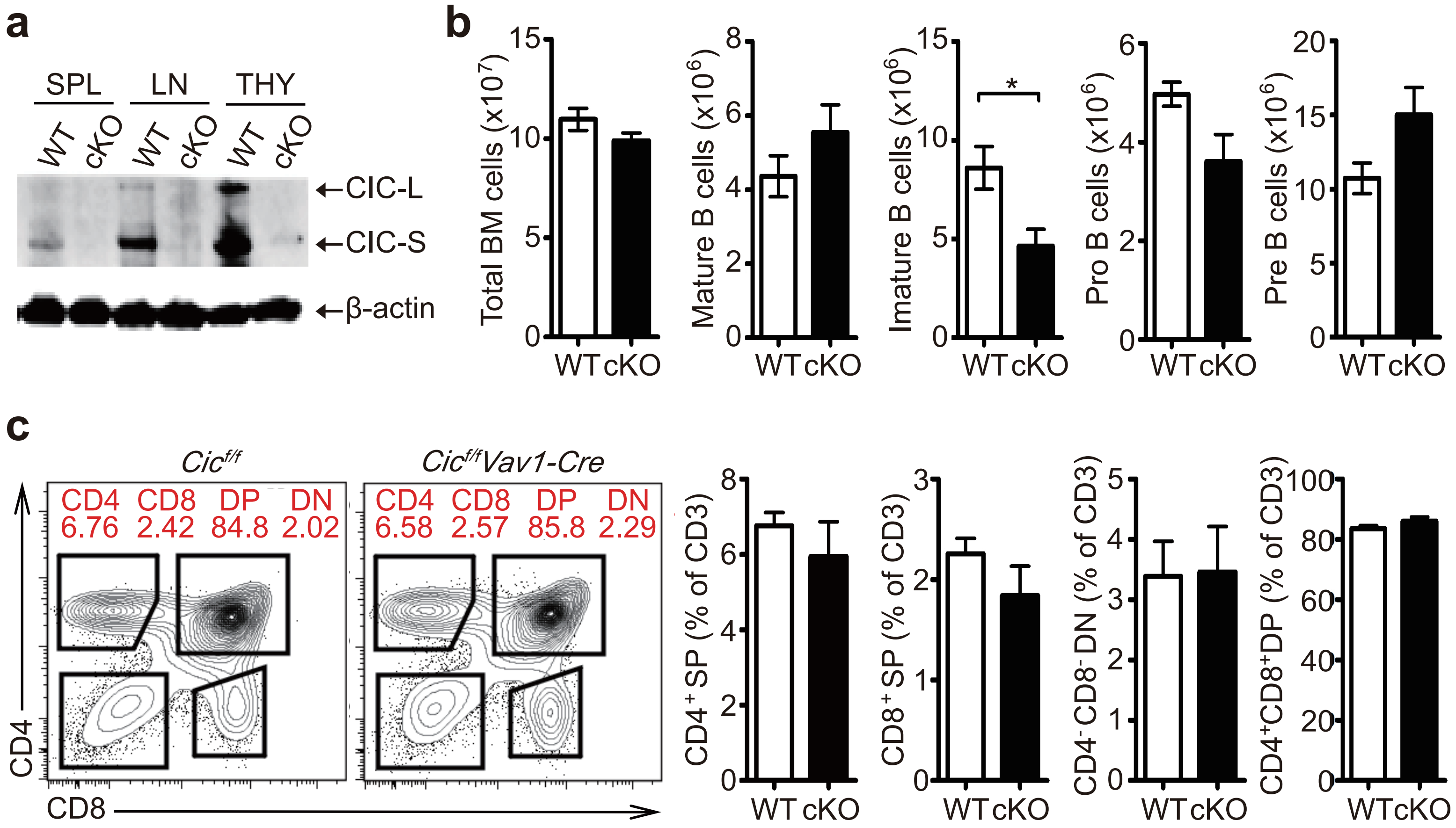

Supplementary Figure 1. Normal development of B and T cells in *Cic^f/f Vav1*-Cre mice.

(**a**) Western blot analysis showing that CIC expression is absent in immune cells from lymphoid organs of *Cic^f/f Vav1*-Cre mice. SPL: spleen, LN: lymph node, THY: thymus. WT and cKO mean *Cic^f/f* and *Cic^f/f Vav1*-Cre mice, respectively.

(**b**) The numbers of total bone marrow cells, mature B cells ($CD43^+IgM^+B220^{hi}$), immature B cells ($CD43^-IgM^+B220^{lo}$), pro-B cells ($CD43^+IgM^-B220^+$) and pre-B cells ($CD43^-IgM^-B220^+$) from *Cic^f/f* and *Cic^f/f Vav1*-Cre mice. All data are representative of two independent experiments with n=4-5 mice per group in each experiment. Error bars indicate SEM.*$p < 0.05$ (two-tailed two-sample unequal variance student t-test).

(**c**) Representative flow cytometry plots (left) and the proportion of thymic CD4SP (CD4 single positive), CD8SP (CD8 single positive), DN (CD4 and CD8 double negative) and DP (CD4 and CD8 double positive) cells in *Cic^f/f* and *Cic^f/f Vav1*-Cre mice. Numbers adjacent to outlined areas indicate percent of CD4SP, CD8SP, DN and DP cells among thymic $CD3^+$ T cells. All data are representative of two independent experiments with n=4-5 mice per group in each experiment. Error bars indicate SEM.

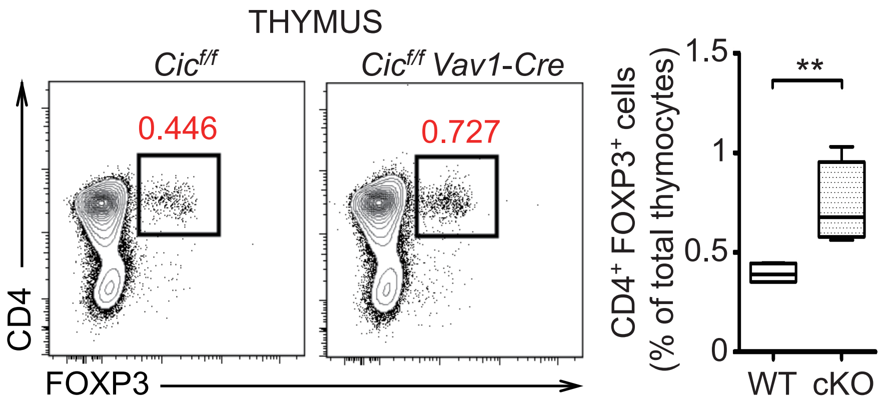

Supplementary Figure 2. Increased proportion of thymic regulatory T cells in *Cic*$^{f/f}$*Vav1*-Cre mice. Representative plots (left) and the percentage (right) of $CD4^+FOXP3^+$ Treg cells in thymus of 9-week-old *Cic*$^{f/f}$ and *Cic*$^{f/f}$*Vav1*-Cre mice. Numbers adjacent to outlined areas indicate percent of $CD4^+FOXP3^+$ cells among thymocytes. Data are representative of two independent experiments with n=5 mice per group in each experiment. The results are presented as box-and-whisker plots. WT: *Cic*$^{f/f}$, cKO: *Cic*$^{f/f}$*Vav1*-Cre. **$p < 0.01$ (two-tailed two-sample unequal variance student t-test).

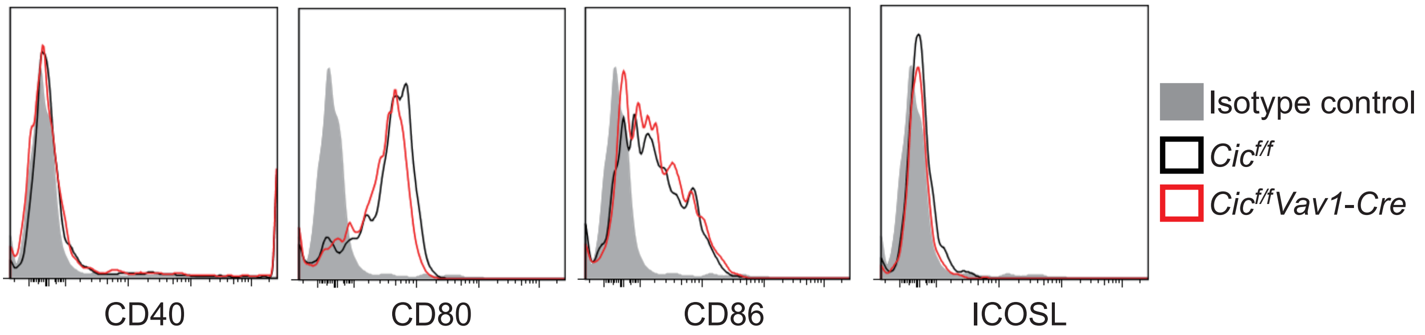

Supplementary Figure 3. Comparable expression of T cell co-stimulatory ligands on the surface of DCs between *Cic*$^{f/f}$ and *Cic*$^{f/f}$*Vav1*-Cre mice.

Comparison of levels of CD40, CD80, CD86 and ICOSL on the surface of CD11b^{+}CD11c^{+}PDCA-1^{-} conventional dendritic cells (cDCs) in spleen between *Cic*$^{f/f}$ and *Cic*$^{f/f}$*Vav1*-Cre mice at 9 weeks of age. Similar results were observed from at least two independent experiments.

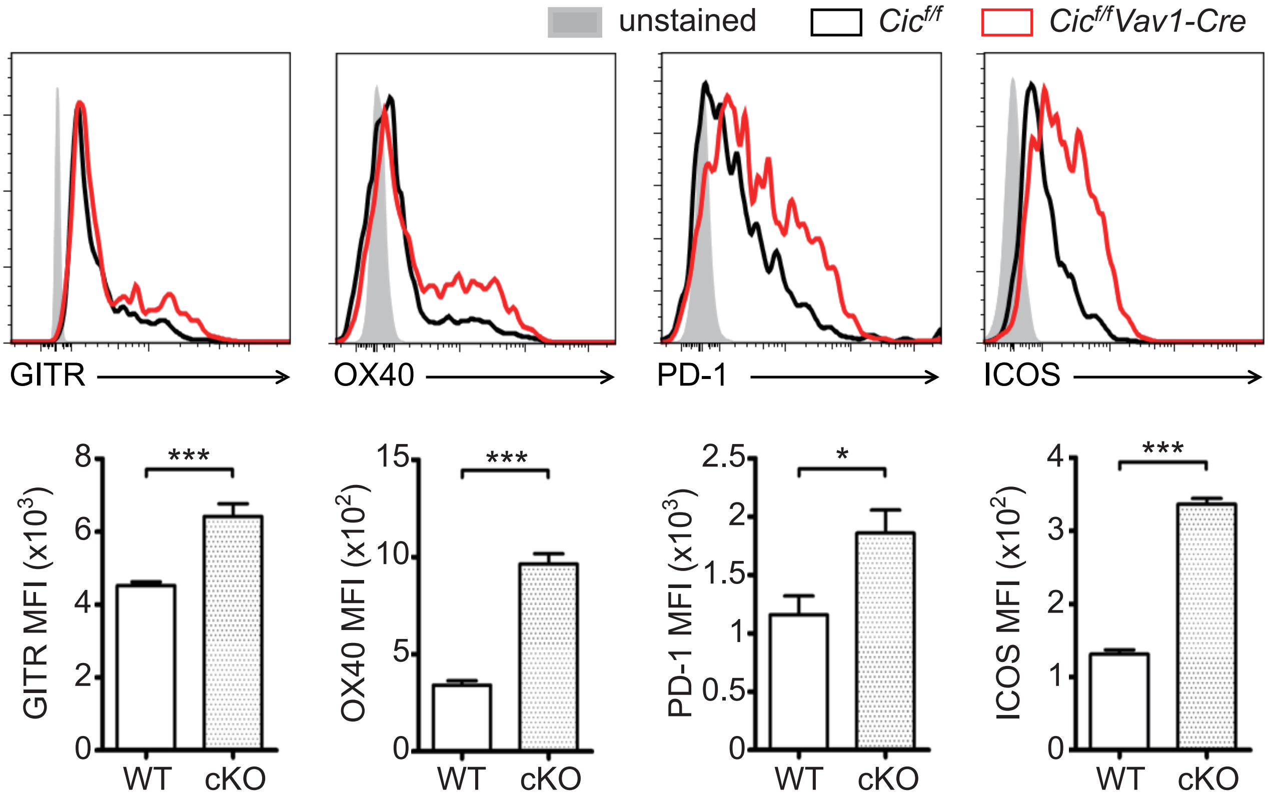

Supplementary Figure 4. Increased surface expression of T cell co-stimulatory molecules on $CD4^+CD44^+$ T cells from *Cic$^{f/f}$Vav1*-Cre mice.

Representative histogram overlay (upper) and mean fluorescence intensity (MFI) (lower) of GITR, OX40, PD-1 and ICOS expressed on $CD4^+CD44^+$ T cells from 9-week-old *Cic$^{f/f}$* and *Cic$^{f/f}$Vav1*-Cre mice. Data are representative of two independent experiments with n=5 mice per group in each experiment. Graphs show data as mean with SEM. *p < 0.05 and ***p < 0.001 (two-tailed two-sample unequal variance student t-test).

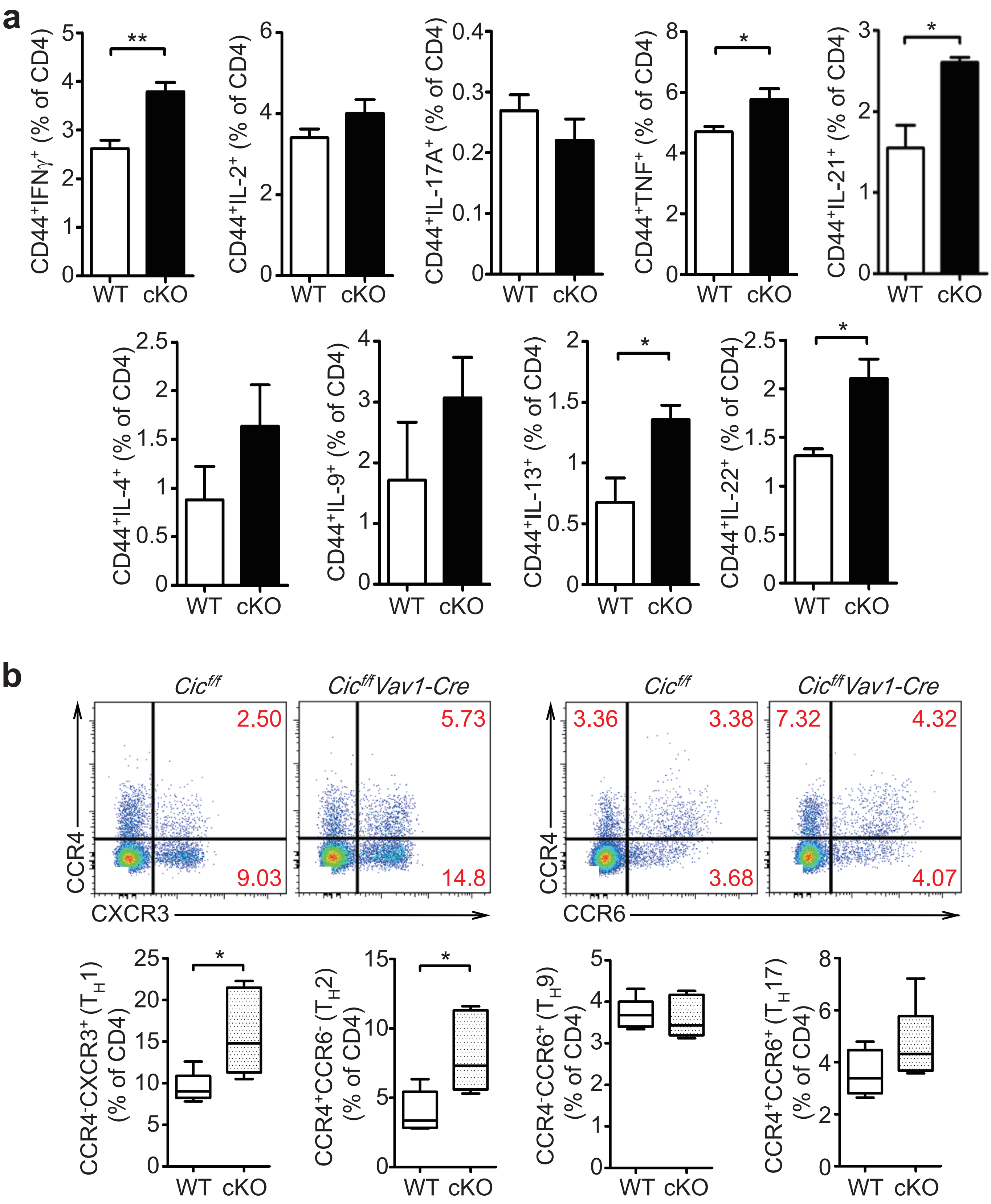

Supplementary Figure 5. Expression profiles of cytokines and chemokine receptors in WT and *Cic* deficient CD4$^+$ T cells.

(**a**) The proportion of various cytokine-expressing CD4$^+$CD44$^+$ T cells in spleen of 9-week-old *Cic$^{f/f}$* and *Cic$^{f/f}$Vav1*-Cre mice. n=3-5 mice per each genotype. Graphs show data as mean with SEM. *p < 0.05 and **p < 0.01 (two-tailed two-sample unequal variance student t-test).

(**b**) FACS analysis for expression profiles of CCR4, CCR6 and CXCR3 on CD4$^+$ T cells in spleen of 9-week -old *Cic$^{f/f}$* and *Cic$^{f/f}$Vav1*-Cre mice. n=5 mice per each genotype. *p < 0.05 (two-tailed two-sample unequal variance student t-test).

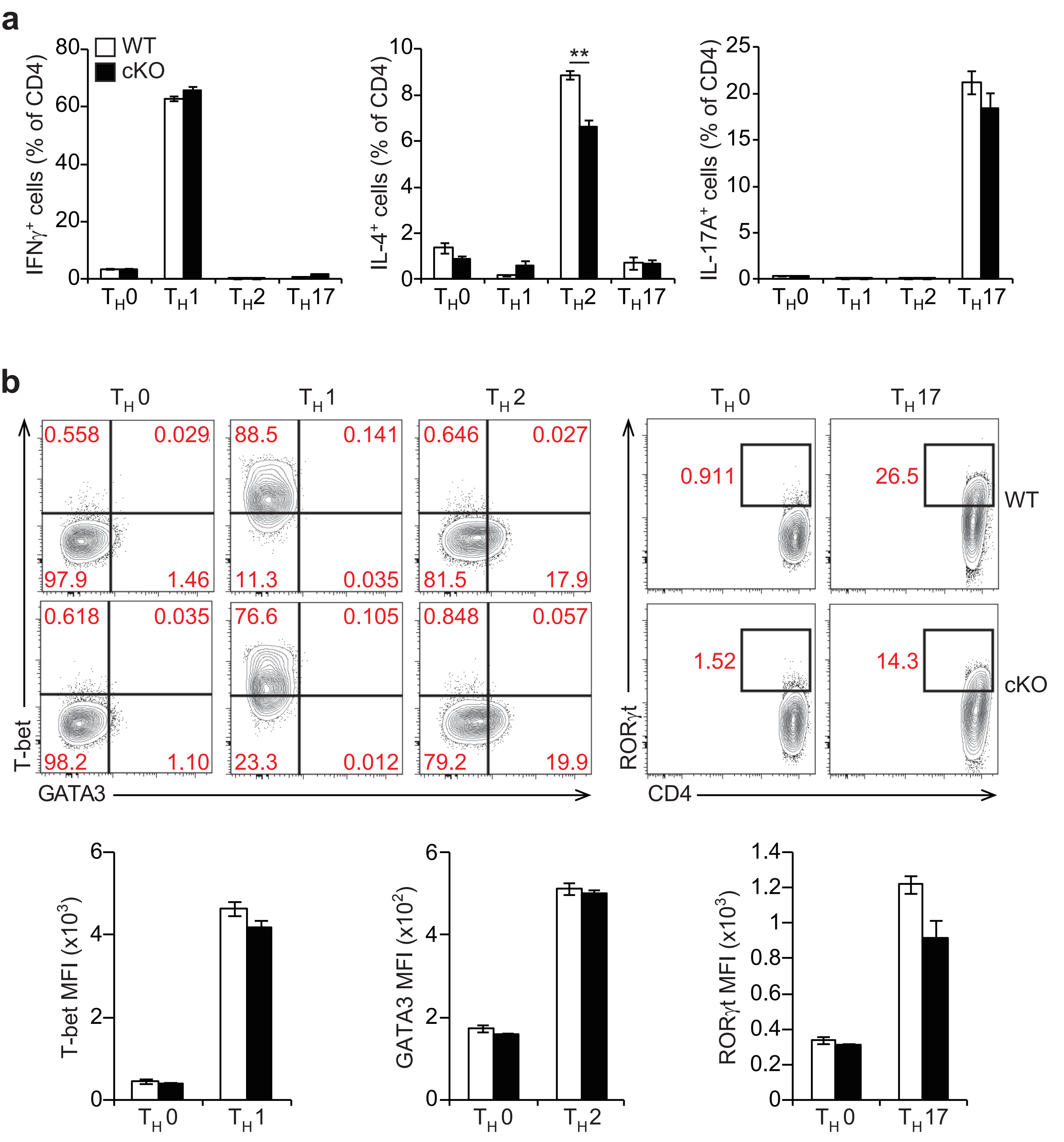

Supplementary Figure 6. Comparable efficiency of T_H1, T_H2 and T_H17 cell differentiation between WT and *Cic* deficient $CD4^+$ T cells.

(**a** and **b**) *In vitro* T helper cell differentiation assay. $CD4^+CD25^-CD44^{lo}CD62L^{hi}$ cells purified from spleens of WT and *Cic* deficient (cKO) mice were differentiated under T_H0, T_H1, T_H2 or T_H17-polarizing condition. The differentiated cells were subjected to intracellular staining of cytokines (**a**) and transcription factors (**b**) followed by flow cytometry analysis. (**a**) Bar graphs for frequency of each cytokine-expressing $CD4^+$ T cells. Three independent experiments were conducted. Error bars indicate SEM. $**p < 0.01$ (two-tailed two-sample unequal variance student t-test). (**b**) Representative FACS plots for the proportion of each transcription factor-expressing $CD4^+$ T cells. Numbers adjacent to outlined areas indicate percent of each cell population. Bar graphs for MFI of each transcription factor expression are presented under the FACS plots. Three independent experiments were performed. Error bars indicate SEM.

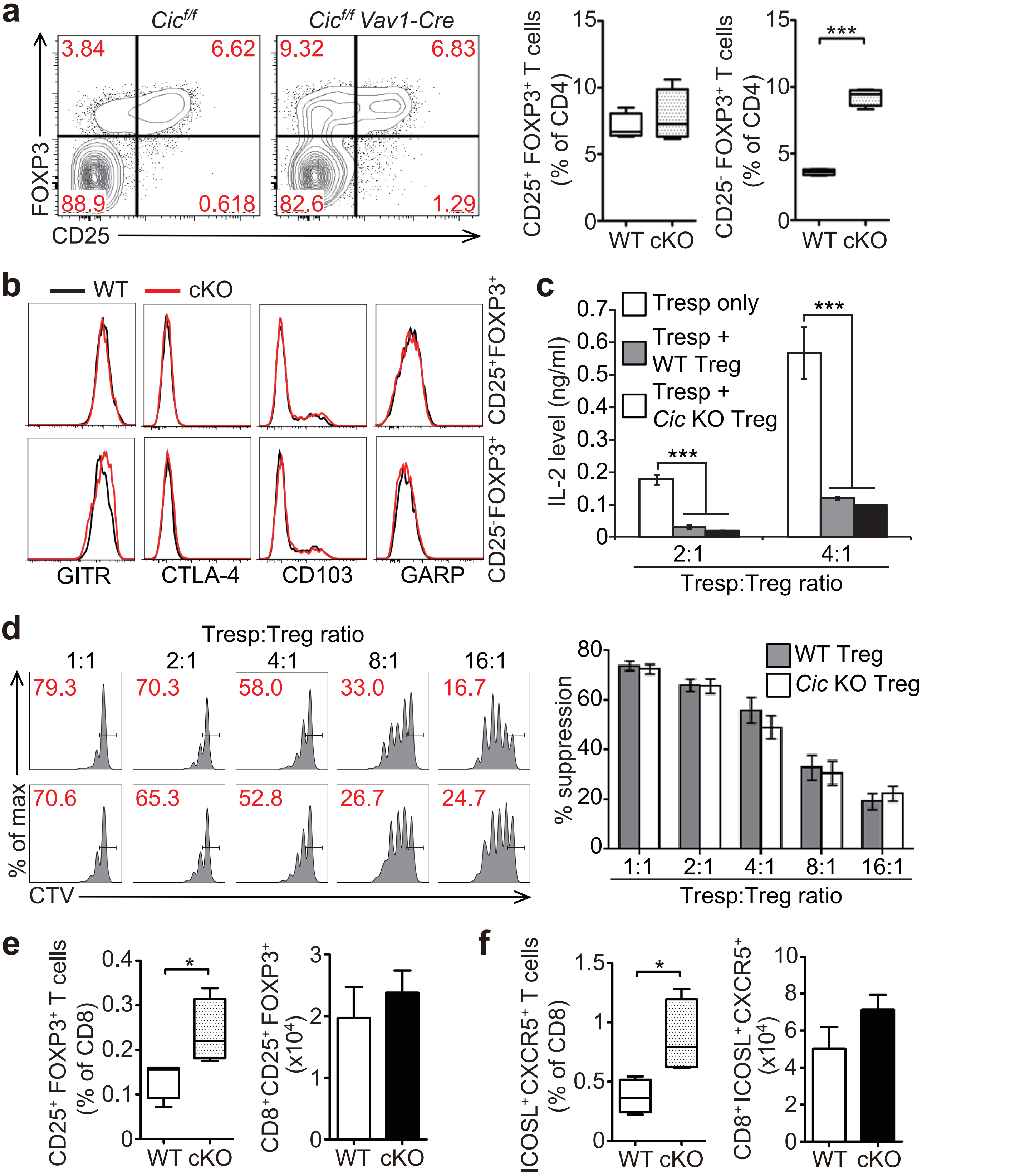

Supplementary Figure 7. Regulatory T cells in spleen of *Cic* deficient mice.

(**a**) Flow cytometry analysis of $CD25^+FOXP3^+$ and $CD25^-FOXP3^+$ $CD4^+$ T cells in spleen of 9-week-old *Cic*$^{f/f}$ (WT) and *Cic*$^{f/f}$*Vav1*-Cre (cKO) mice. Data are representative of two independent experiments with n=4-5 mice per group in each experiment. ***$p < 0.001$ (two-tailed two-sample unequal variance student t-test).

(**b**) Representative histogram overlays for expression of co-stimulatory molecules on $CD25^+FOXP3^+$ and $CD25^-FOXP3^+$ $CD4^+$ T cells from WT and *Cic* deficient mice.

(**c** and **d**) Treg cell suppression assay. (**c**) $CD4^+CD25^-$ responder cells (Tresp) were stimulated with plate-bound anti-CD3 (1μg/ml) and anti-CD28 (1μg/ml) in the presence or absence of $CD4^+CD25^+$ Treg cells from WT and *Cic* deficient mice. Concentration of IL-2 in culture media was determined by ELISA. n=3 per each sample. (**d**) Flow cytometry analysis of CTV dilution in Tresp cells cultured with increasing numbers of Treg cells from WT and *Cic* deficient mice. The numbers in histograms indicate the percentage of non-proliferative cell population among the Tresp cells. The bar graph shows data as mean±SEM.

(**e** and **f**) The frequency and the number of $CD8^+$ regulatory T cells ($CD25^+FOXP3^+$ (**e**) and $ICOSL^+CXCR5^+$ (**f**) $CD8^+$ T cells) in spleen of 9-week-old *Cic*$^{f/f}$ and *Cic*$^{f/f}$*Vav1*-Cre mice. n=4 mice per each genotype. *$p < 0.05$.

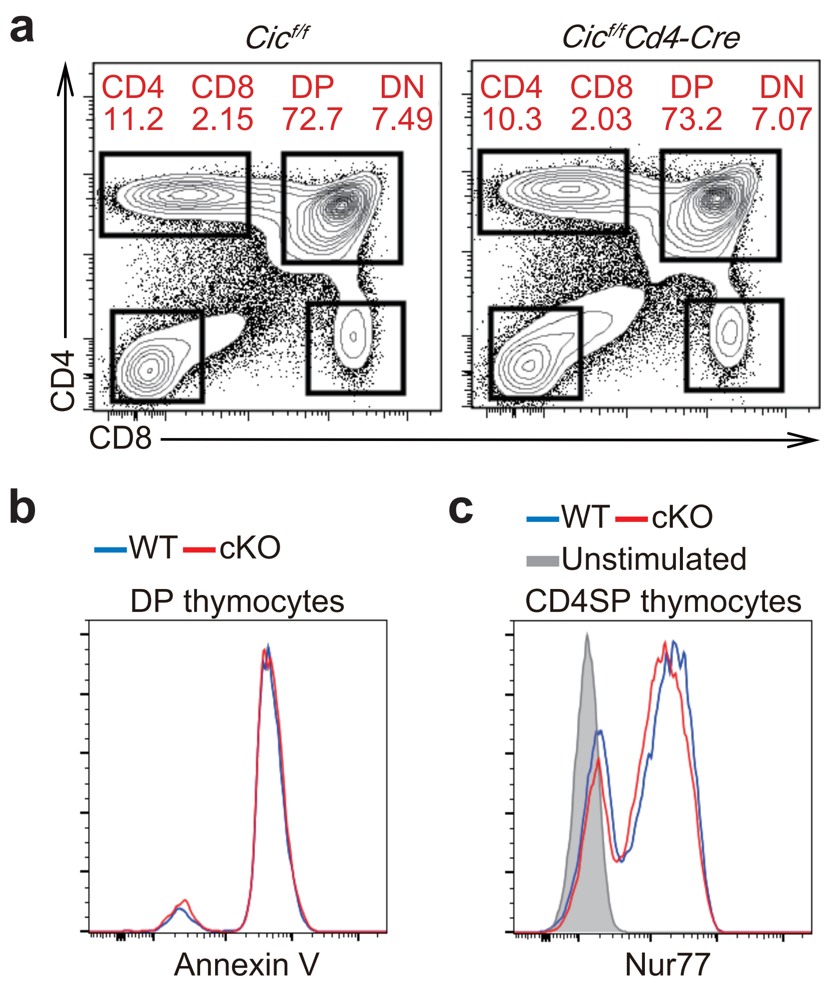

Supplementary Figure 8. Normal thymic development and negative selection of T cells in *Cic*$^{f/f}$*Cd4*-Cre mice.
(**a**) Representative FACS plots for the proportions of thymic CD4SP, CD8SP, DN and DP cells among thymocytes of 12-week-old *Cic*$^{f/f}$ and *Cic*$^{f/f}$*Cd4*-Cre mice.
(**b**) A representative histogram overlay for TCR-induced cell death assay. Sorted DP thymocytes from *Cic*$^{f/f}$ (WT) and *Cic*$^{f/f}$*Cd4*-Cre (cKO) mice were stimulated with anti-CD3 and anti-CD28 for 24 h. Cell death was assessed by staining for CD4, CD8 and Annexin V followed by flow cytometry analysis.
(**c**) A representative histogram overlay for induction of Nur77 expression in WT and *Cic* null CD4SP thymocytes upon TCR stimulation. Thymocytes were stimulated with anti-CD3 and anti-CD28 for 6 h and then subjected to intracellular staining for Nur77. (**b** and **c**) Similar results were observed from two independent expriments.

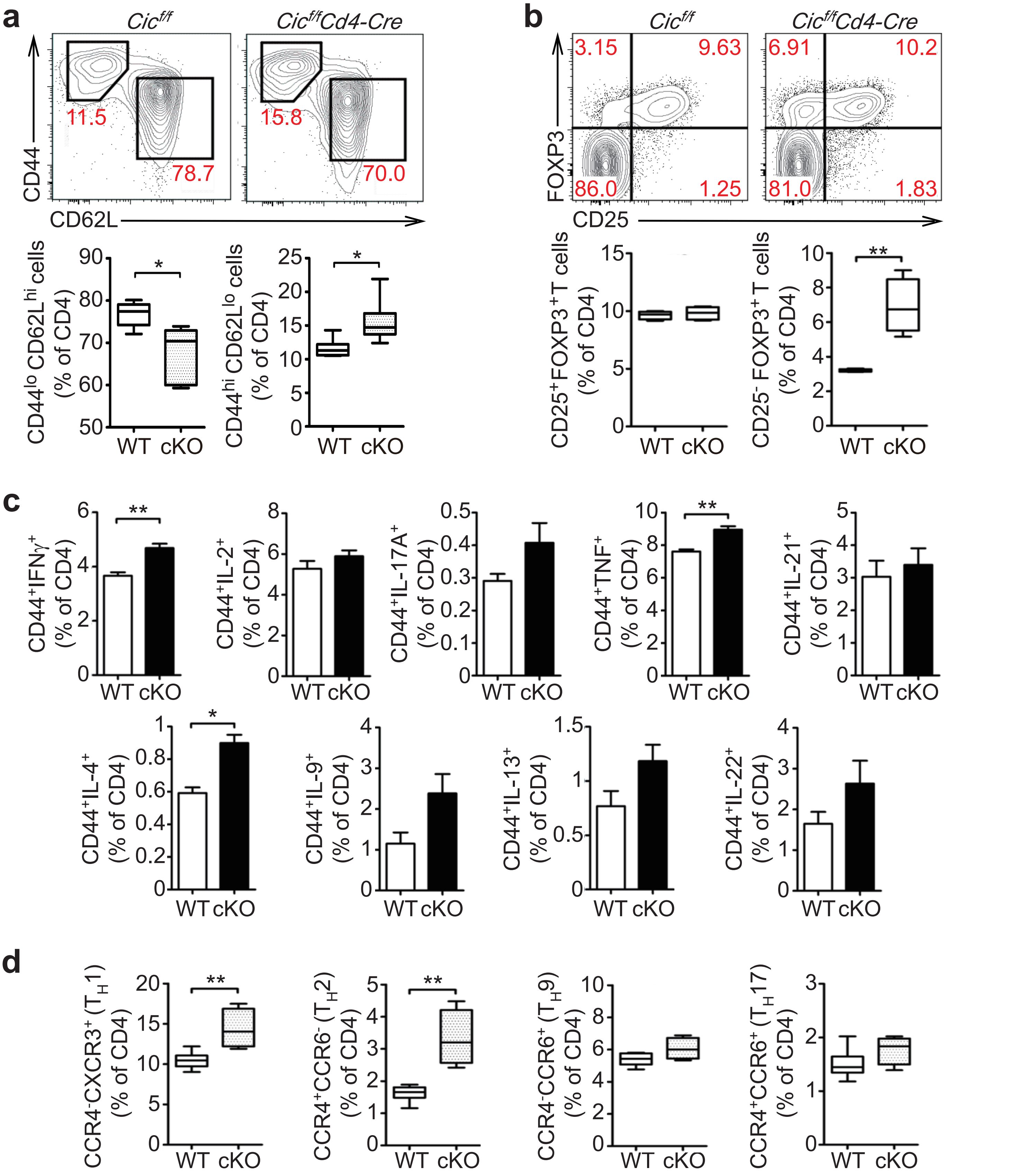

Supplementary Figure 9. Hyperactivation of CD4+ T cells and increased proportion of CD25-FOXP3+, T_H1 and T_H2 cells in spleen of *Cic*$^{f/f}$*Cd4*-Cre mice.

(**a** and **b**) Flow cytometry analyses of naïve ($CD44^{lo}CD62L^{hi}$) and effector/memory ($CD44^{hi}CD62L^{lo}$) $CD4^+$ T cells (**a**), and $CD25^+FOXP3^+$ and $CD25^-FOXP3^+$ $CD4^+$ T cells (**b**) in spleen of 12-week-old *Cic*$^{f/f}$ (WT) and *Cic*$^{f/f}$*Cd4*-Cre (cKO) mice. All data are representative of two independent experiments with n=4-5 mice per group in each experiment.

(**c** and **d**) Flow cytometry analysis for expression profiles of cytokines (**c**) and chemokine receptors (**d**) on $CD4^+$ T cells in spleen of 12-week-old *Cic*$^{f/f}$ and *Cic*$^{f/f}$*Cd4*-Cre mice. (**c**) n=3 mice per each genotype. (**d**) n=4-6 mice per each genotype. Error bars indicate SEM. *$p < 0.05$ and **$p < 0.01$ (two-tailed two-sample unequal variance student t-test).

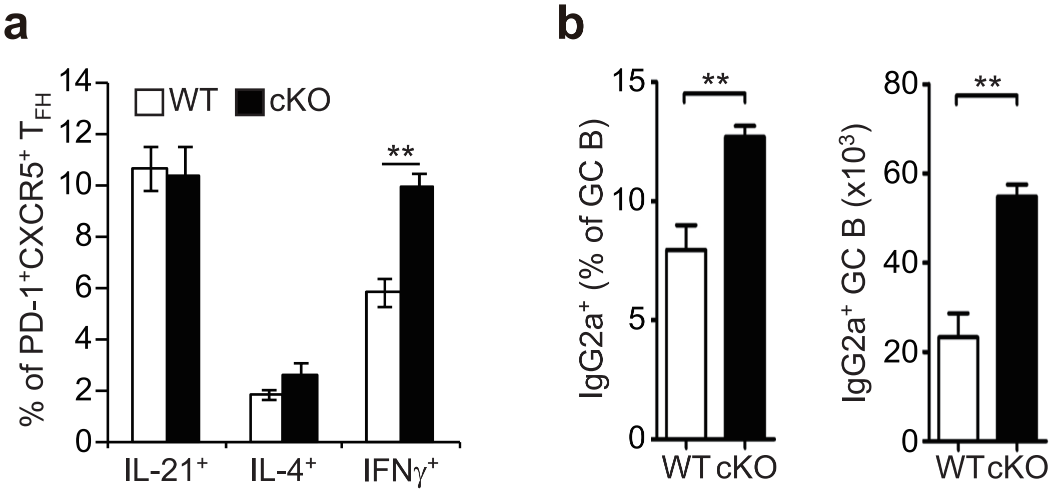

Supplementary Figure 10. Increased frequency of IFNγ$^+$ T$_{FH}$ and IgG2a$^+$ GC B cells in spleen of *Cic$^{f/f}$Cd4*-Cre mice.

(**a**) Flow cytometry analysis of IL-21-, IL-4- or IFNγ-expressing T$_{FH}$ cells in spleen of 12-week-old *Cic$^{f/f}$* (WT) and *Cic$^{f/f}$Cd4*-Cre (cKO) mice. The proportions of each cytokine-expressing T$_{FH}$ cells are presented.
(**b**) Flow cytometry analysis of IgG2a$^+$ GC B cells in spleen of about 5-month-old *Cic$^{f/f}$* and *Cic$^{f/f}$Cd4*-Cre mice. The proportion (left graph) and the number (right graph) of IgG2a$^+$ GC B cells are presented.
Error bars indicate SEM. **$p < 0.01$ (two-tailed two-sample unequal variance student t-test).

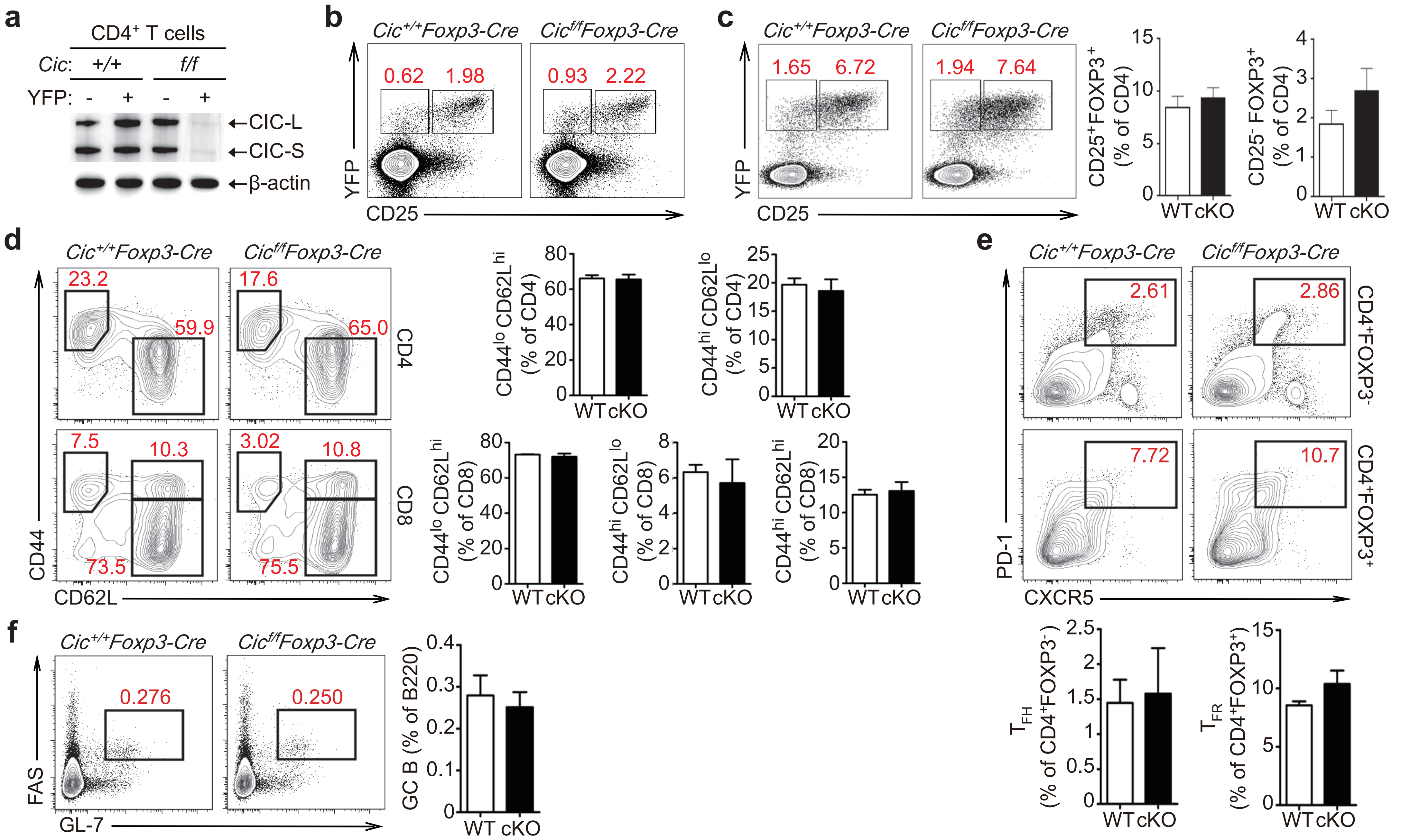

Supplementary Figure 11. Normal Treg cell development, pheripheral T cell homeostasis and GC response in Treg cell-specific *Cic* null mice. (**a**) Western blot analysis showing Treg cell-specific ablation of CIC expression in *Cic*$^{f/f}$*Foxp3*-YFP-Cre mice. $CD4^{+}YFP^{+}$ and $CD4^{+}YFP^{-}$ T cells were purified from secondary lymphoid organs of 18-week-old *Cic*$^{+/+}$*Foxp3*-YFP-Cre (WT) and *Cic*$^{f/f}$*Foxp3*-YFP-Cre (cKO) mice. (**b** and **c**) Flow cytometry analyses of $CD4^{+}$ Treg cells in thymus (**b**) and spleen (**c**) of 9-week-old WT and cKO mice. WT, n=5; cKO, n=4. (**d**-**f**) Flow cytometry analyses of naïve ($CD44^{lo}CD62L^{hi}$) and effector/memory ($CD44^{hi}CD62L^{lo}$) $CD4^{+}$ T cells (**d**), T_{FH} and T_{FR} cells (**e**) and GC B cells (**f**) in spleen of 9-week-old WT and cKO mice. WT, n=5; cKO, n=3. Error bars indicate SEM.

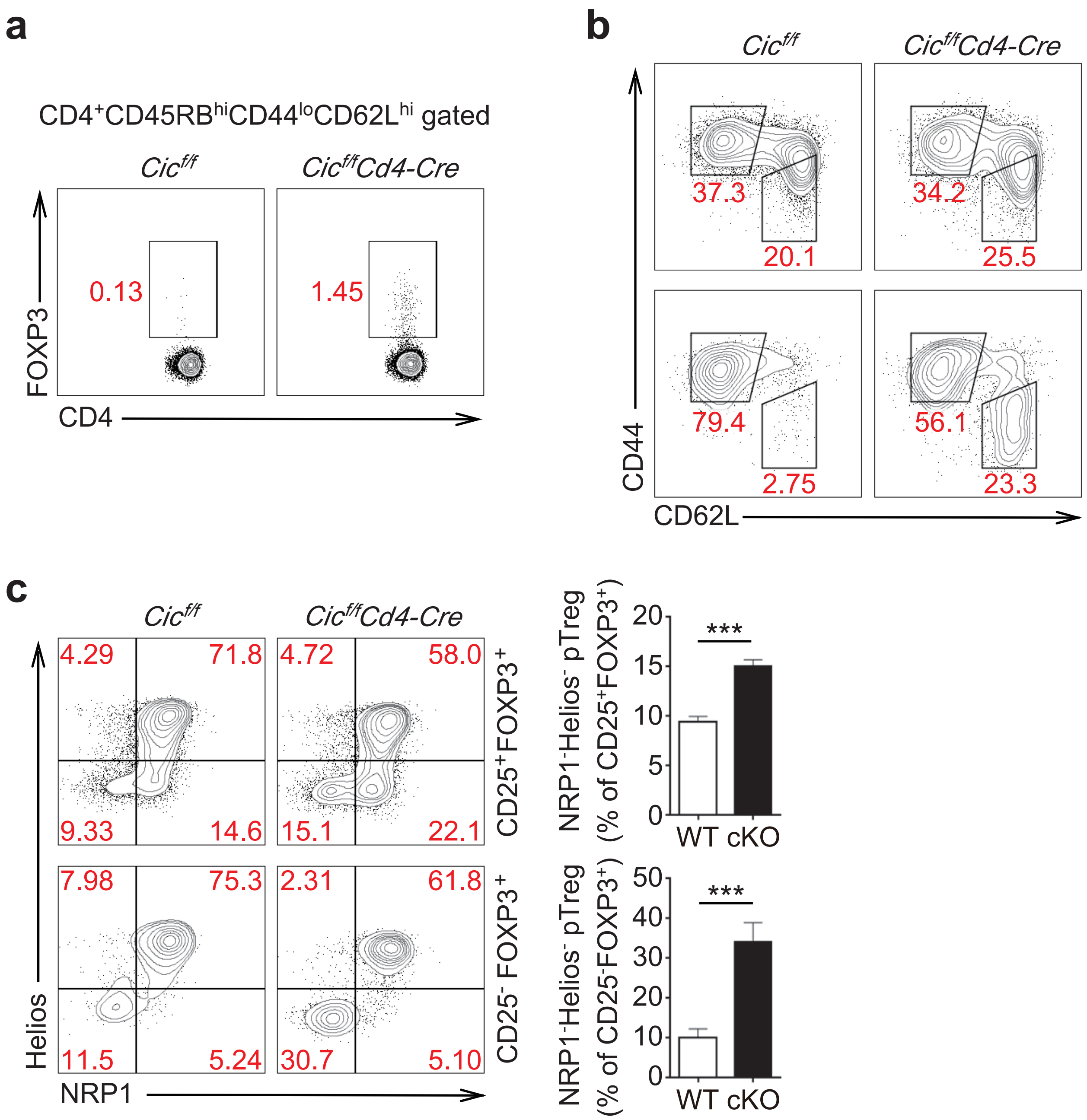

Supplementary Figure 12. Increased frequency of FOXP3-expressing naive CD4$^+$ T cells and pTreg cells in spleen of *Cic$^{f/f}$Cd4-Cre mice.*

(**a**) Flow cytometry analysis of FOXP3-expressing naive (CD45RB^{hi}CD44^{lo}CD62L^{hi}) CD4$^+$ T cells in spleen of 12-week-old *Cic$^{f/f}$* and *Cic$^{f/f}$Cd4*-Cre mice.

(**b**) Flow cytometry analysis for expression of CD44 and CD62L on splenic CD25$^+$FOXP3$^+$ and CD25$^-$FOXP3$^+$ CD4$^+$ T cells from 12-week-old *Cic$^{f/f}$* and *Cic$^{f/f}$Cd4*-Cre mice. The frequency of CD4$^+$CD25$^-$FOXP3$^+$ T cells with naive (CD44^{lo}CD62L^{hi}) phenotype was dramatically increased in *Cic$^{f/f}$Cd4*-Cre mice.

(**c**) Flow cytometry analysis for expression of Helios and Neurophilin-1 (NRP1) on splenic CD25$^+$FOXP3$^+$ and CD25$^-$FOXP3$^+$ CD4$^+$ T cells from 12-week-old *Cic$^{f/f}$* (WT) and *Cic$^{f/f}$Cd4*-Cre (cKO) mice. The proportions of CD25$^+$FOXP3$^+$ and CD25$^-$FOXP3$^+$ NRP1$^-$Helios$^-$ pTreg cells were significantly increased in *Cic$^{f/f}$Cd4*-Cre mice. WT, n=6; cKO, n=5. Error bars indicate SEM. ***p < 0.001 (two-tailed two-sample unequal variance student t-test).

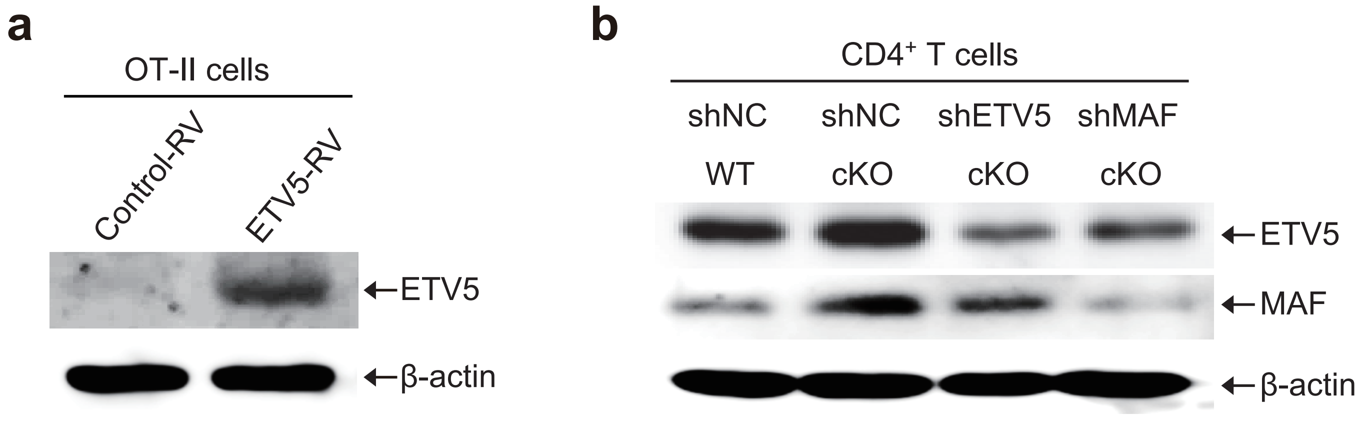

Supplementary Figure 13. Overexpression of ETV5 and knockdown of ETV5 and MAF in CD4$^+$ T cells.

(**a**) Western blot analysis showing overexpression of ETV5 in OT-II T cells transduced with ETV5 expressing retrovirus.

(**b**) Western blot analysis showing knockdown efficiency of shRNAs for *Etv5* (shETV5) and *Maf* (shMAF). CD4$^+$ T cells purified from spleens of *Cic*$^{f/f}$ (WT) and *Cic*$^{f/f}$*Cd4*-Cre (cKO) mice were transduced with control (shNC), shETV5 or shMAF expressing retrovirus. GFP$^+$ cells (about 20~30%) were FACS-sorted and subjected to western blot analysis for ETV5 and MAF.

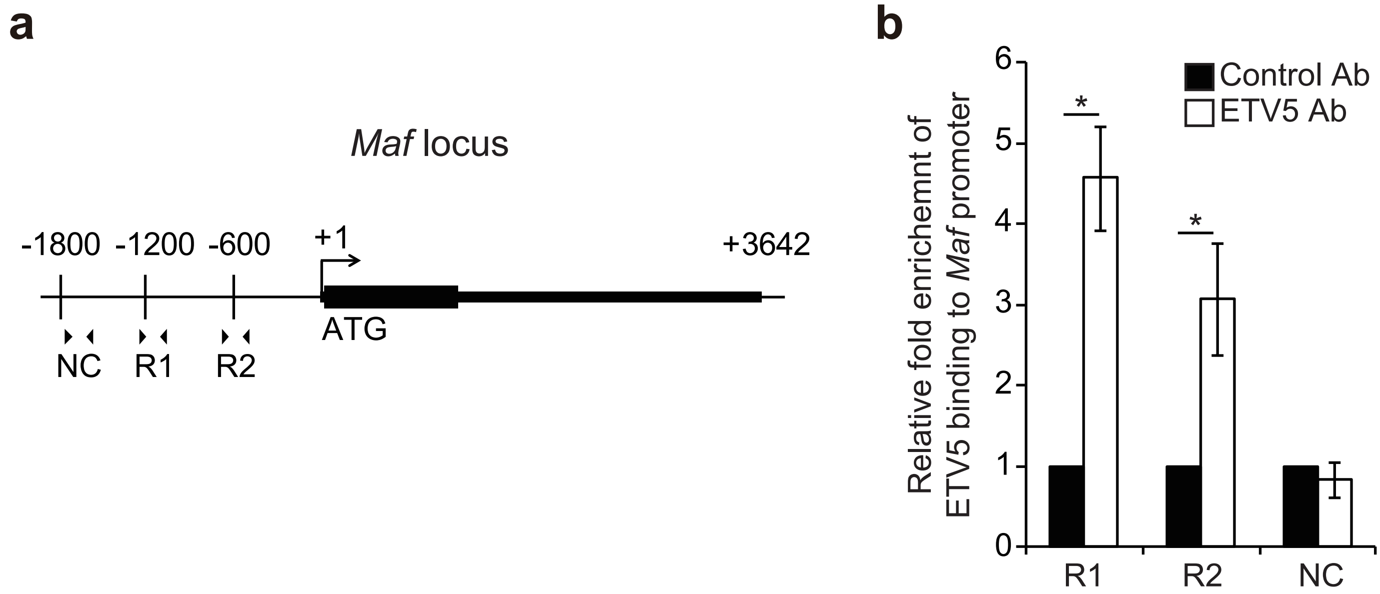

Supplementary Figure 14. Direct binding of ETV5 to *Maf* promoter in CD4⁺ T cells.
(**a**) Schematic illunstration of mouse *Maf* locus (RefSeq: NM_001025577.2). Two different *Maf* promoter regions (R1: -1240/-976 and R2: -697/-532), which were previously validated as ETV5 binding regions (Xie et al. 2016), and negative control (NC) region (-1768/-1520) that does not contain Ets consensus sequence (CCGGA(A/T)(A/G)(C/T)) are marked by arrow heads.
(**b**) ChIP-qPCR analysis showing ETV5 promoter occupancy of *Maf* in $CD4^+$ T cells. The $CD4^+$ T cells activated with anti-CD3 and anti-CD28 in the presence of IL-6 were subjected to ChIP experiment using anti-ETV5 antibody (ETV5 Ab). Anti-GFP antibody was used as a negative control antiboby (Control Ab). Input and antiboby-associated DNA fragments were then subjected to qPCR analysis for each *Maf* promoter region (R1, R2 and NC). More than three independent experiments were performed. Error bars indicate SEM.
*$p < 0.05$ (two-tailed two-sample unequal variance student t-test).

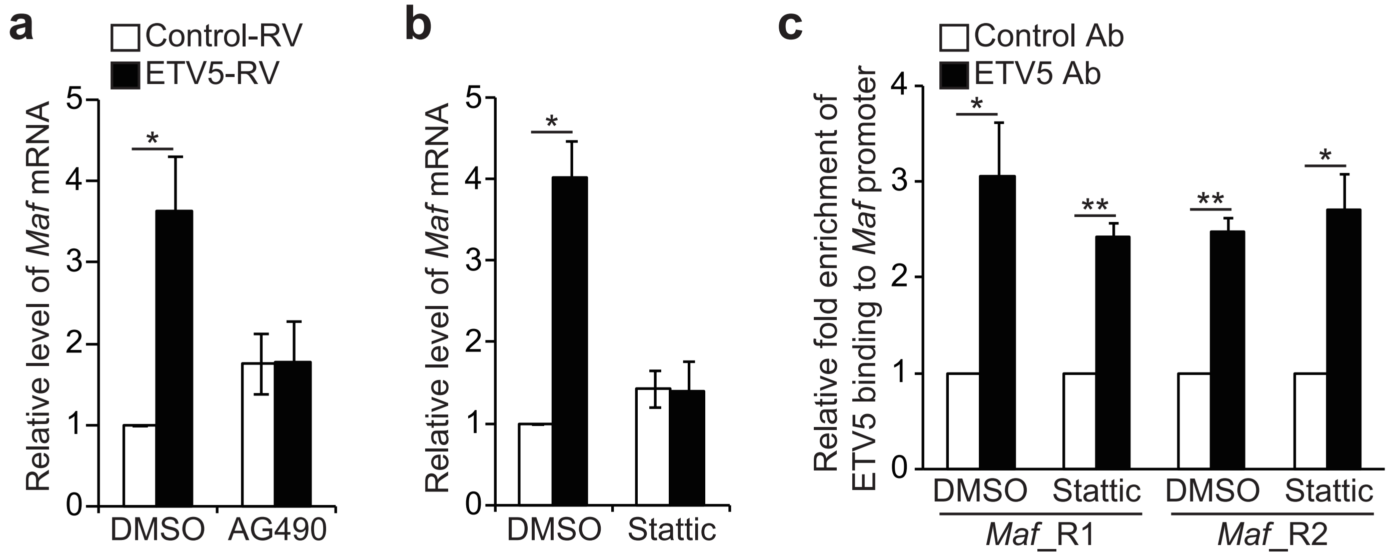

Supplementary Figure 15. JAK2-STAT3 pathway is involved in ETV5-mediated induction of *Maf* expression.

(**a** and **b**) qRT-PCR analysis for *Maf* levels. $CD4^+$ T cells infected with control or ETV5-expressing retrovirus were treated with AG490 (JAK2 inhibitor) (**a**) or Stattic (STAT3 inhibitor) (**b**) and cultured under T_{FH}-like condition (IL-6 and IL-21). Total RNA was extracted from the cells and subjected to qRT-PCR analysis for *Maf* levels. Inhibition of JAK2 and STAT3 completely suppressed induction of *Maf* expression by ETV5 overexpression in $CD4^+$ T cells. Three independent experiments were carried out.

(**c**) ChIP-qPCR analysis for ETV5 binding to *Maf* promoter in $CD4^+$ T cells treated with Stattic. The two Ets consensus sequence-containing *Maf* promoter regions (R1 and R2) were examined. Inhibition of STAT3 did not affect ETV5 binding to *Maf* promoter. Four independent experiments were carried out. Error bars indicate SEM. *p<0.05 and **p<0.01 (two-tailed two-sample unequal variance student t-test).

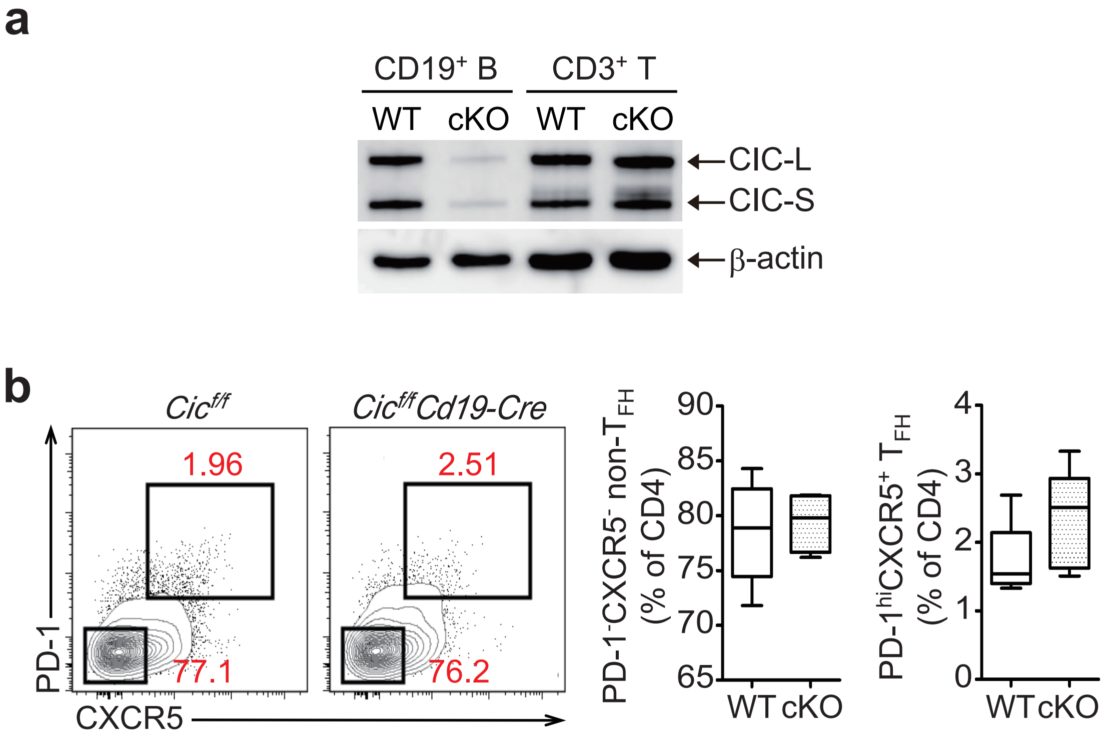

Supplementary Figure 16. Normal T_{FH} cell formation in B cell-specific *Cic* null mice.
(**a**) Western blot analysis for levels of CIC in B and T cells from *Cic*$^{f/f}$ (WT) and *Cic*$^{f/f}$*Cd19*-Cre (cKO) mice. FACS-sorted CD19^{+} B and CD3^{+} T cells were subjected to western blot analysis for CIC.
(**b**) Comparable frequency of splenic CD4^{+}PD-1^{hi}CXCR5^{+} T_{FH} cells between *Cic*$^{f/f}$ and *Cic*$^{f/f}$*Cd19*-Cre mice at 12 weeks of age. n=4 mice per each genotype.

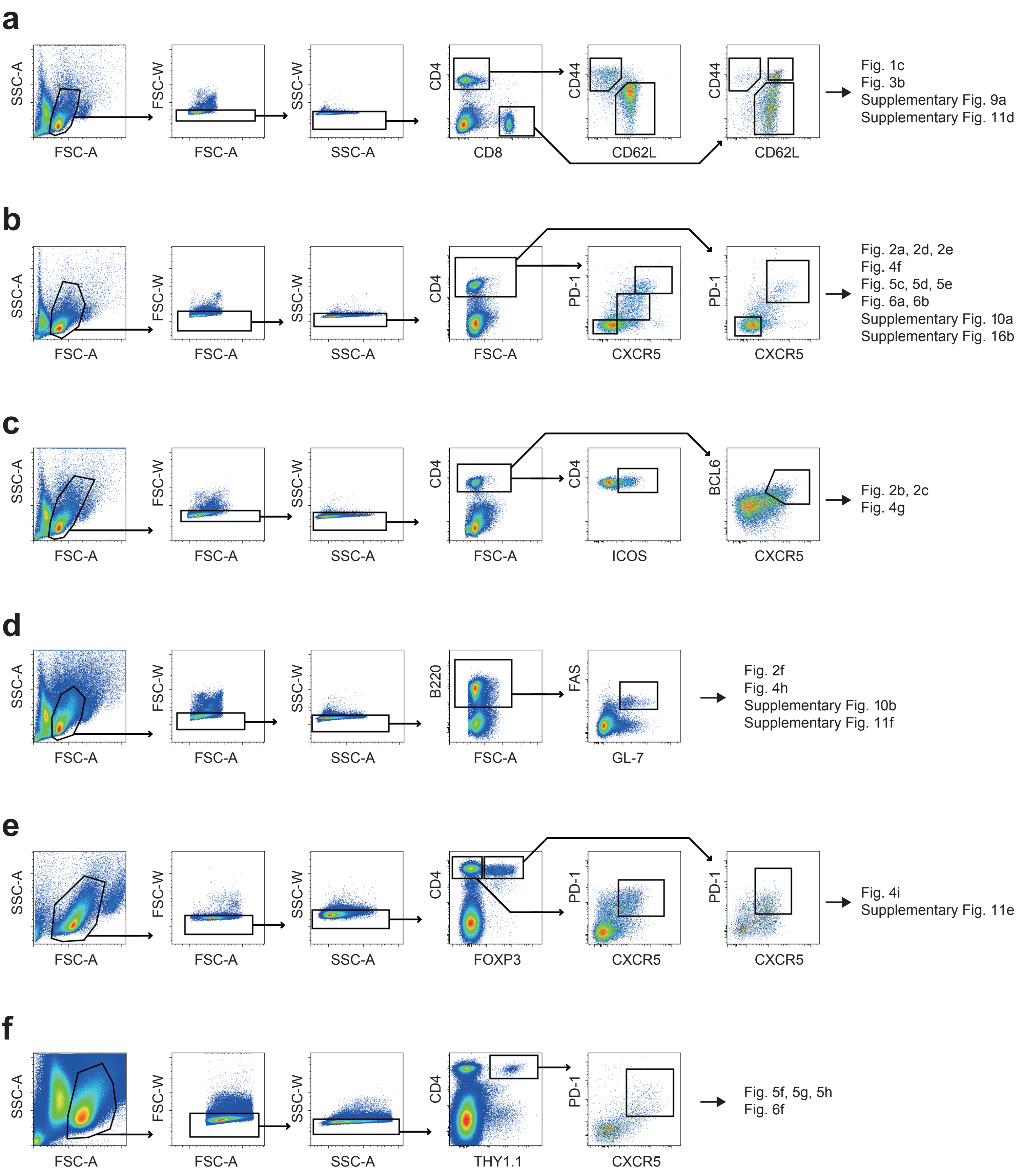

Supplementary Figure 17. Flow cytometry gating strategies.
(**a**) Sequential gating strategy for naïve and effector/memory T cell populations in splenocytes.
(**b**) Sequential gating strategy for $CD4^+$ T cells expressing PD-1 and CXCR5.
(**c**) Sequential gating strategy for $CD4^+$ T cells expressing ICOS, BCL6, and CXCR5.
(**d**) Sequential gating strategy for $B220^+$ B cells expressing FAS and GL-7.
(**e**) Sequential gating strategy for $CD4^+$ T cells expressing FOXP3, PD-1, and CXCR5.
(**f**) Sequential gating strategy for THY1.1$^+$ $CD4^+$ T cells expressing PD-1 and CXCR5.

Continued Supplementary Figure 17

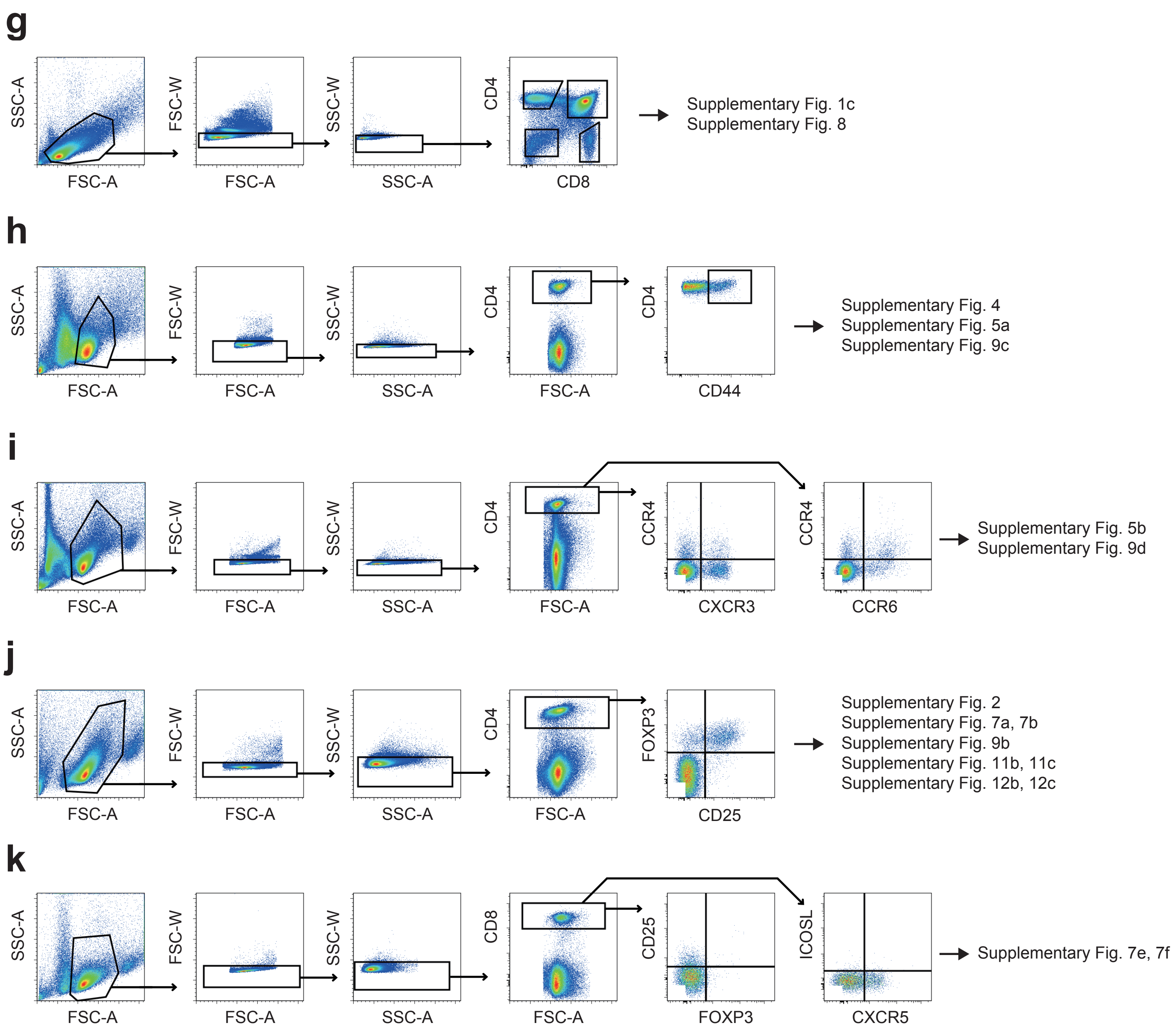

Supplementary Figure 17. Flow cytometry gating strategies (Continued).
(**g**) Sequential gating strategy for SP, DP, and DN populations in thymocytes.
(**h**) Sequential gating strategy for CD4$^+$ CD44$^+$ T cells.
(**i**) Sequential gating strategy for CD4$^+$ T cells expressing CCR4, CCR6, and CXCR3.
(**j**) Sequential gating strategy for CD4$^+$ T cells expressing FOXP3 and CD25.
(**k**) Sequential gating strategy for CD25$^+$FOXP3$^+$ and ICOSL$^+$CXCR5$^+$ CD8$^+$ T cells.

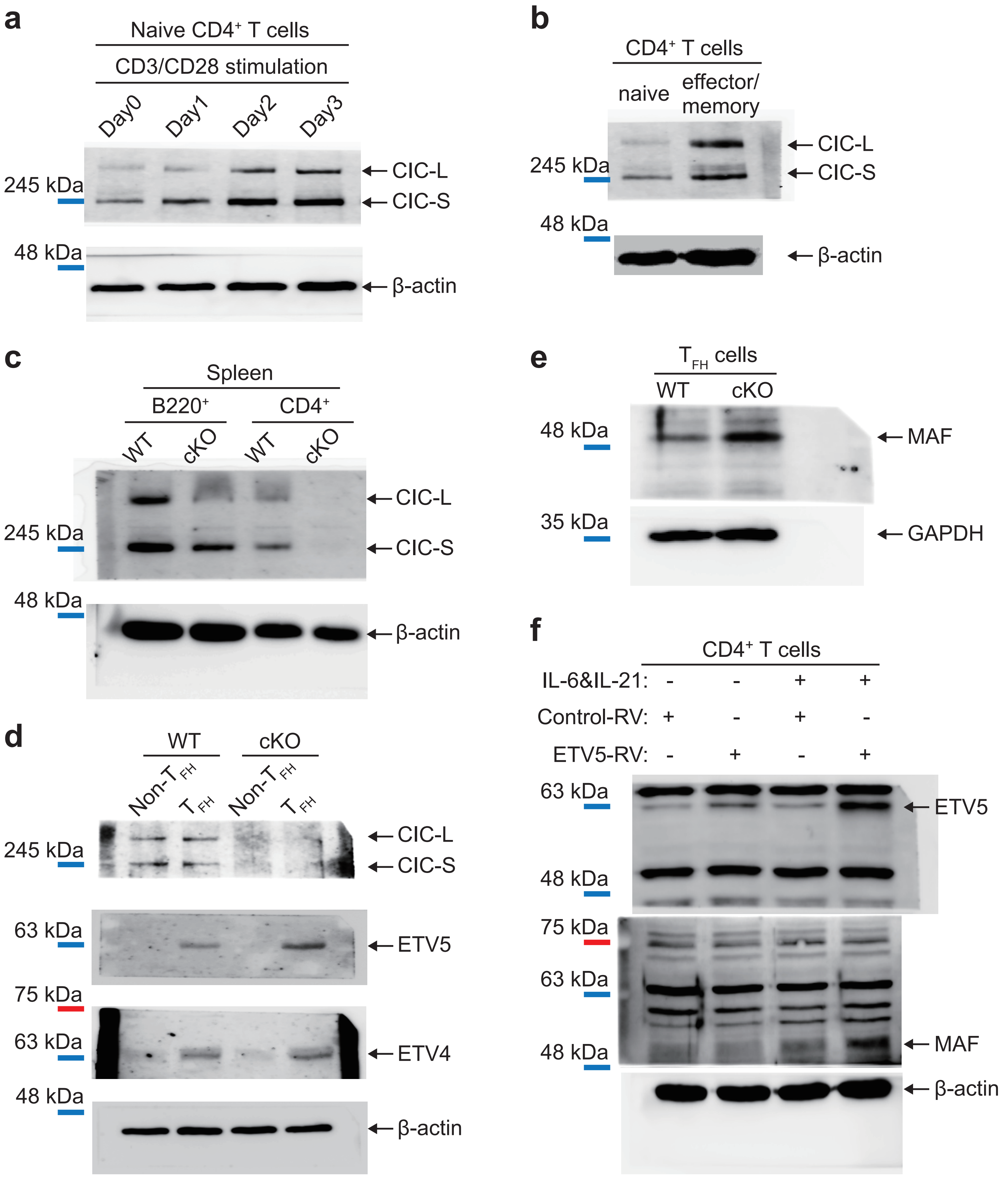

Supplementary Figure 18. Raw western blot images used in main figures.
(**a-f**) The full western blot images of Fig. 3a (**a**), Fig. 3b (**b**), Fig. 4a (**c**), Figs. 5d and 5e (**d**), Fig. 6b (**e**) and Fig. 6d (**f**).

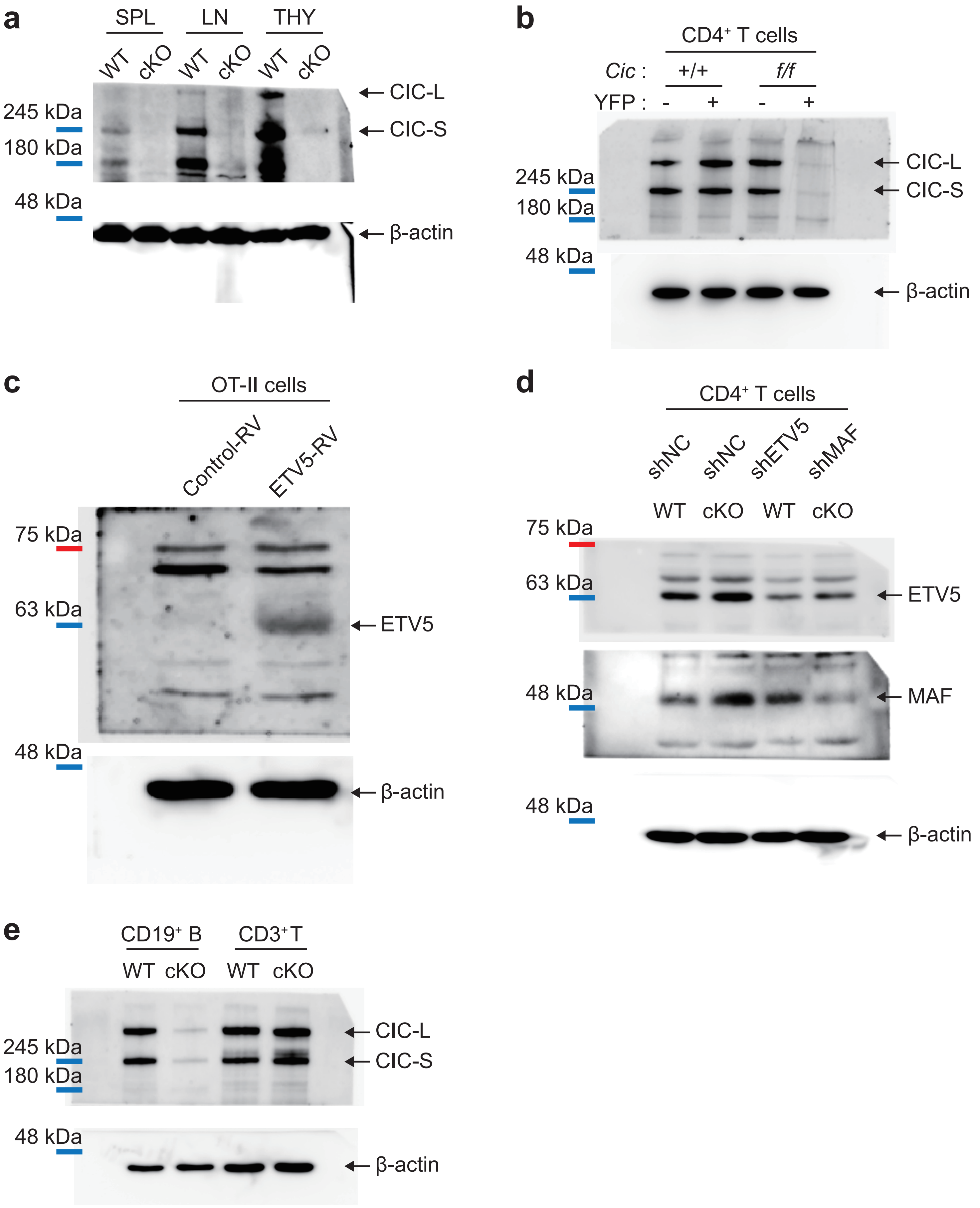

Supplementary Figure 19. Raw western blot images used in supplementary figures.
(**a**-**e**) The full western blot images of Supplementary Fig. 1a (**a**), Fig. 11a (**b**), Fig. 13a (**c**), Fig. 13b (**d**) and Fig.16a (**e**).
